# Surface Anatomy and Sensory Evaluation of Dermatomes: A Guide for Residents

**DOI:** 10.21315/mjms-09-2024-738

**Published:** 2025-02-28

**Authors:** Balamurugan Rajendran, Muhammad Asyraf Yunos, Harivarmah Nagalinggam, Mohamad Lokman Abdul Aziz, Jafri Malin Abdullah

**Affiliations:** 1 Department of Neurosciences, School of Medical Sciences, Universiti Sains Malaysia, Health Campus, Kelantan, Malaysia; 2 Hospital Pakar Universiti Sains Malaysia, Universiti Sains Malaysia, Health Campus, Kelantan, Malaysia; 3 Department of Neurosurgery, Hospital Raja Permaisuri Bainun, Ipoh, Perak, Malaysia; 4 Brain and Behaviour Cluster, School of Medical Sciences, Universiti Sains Malaysia, Health Campus, Kelantan, Malaysia

**Keywords:** dermatome, sensory, surface anatomy

## Abstract

The surface anatomy of dermatomes and sensory examination play crucial roles in assessing and diagnosing various medical conditions. Understanding the distribution of dermatomes and conducting sensory examinations are essential in identifying and localising neurological disorders, such as nerve damage or compression. This manuscript describes the surface anatomy of the face and body and their respective key sensory examination points for sensory and neurological examination to aid Malaysian medical students and trainees in lesion localisation.

## Introduction

Sensory examination is critical in neurological examination and diagnostic assessment, particularly in lesion localisation (1). The primary aim of sensory testing is to validate sensory integrity and to unravel the complex interactions between the central nervous system and peripheral nervous system, thereby guiding us in localising the lesion (2).

The skin is divided into several areas known as dermatomes. Dermatomes, which are specific areas of skin innervated by sensory nerves emerging from a single spinal nerve root (3, 4), are the cornerstone of sensory testing. During vertebrate development, the somite gives rise to the sclerotome, myotome, and dermatome (3). The sclerotome develops into the vertebrae, the myotome into the skeletal muscle, and the dermatome into the skin (3). Each dermatome comprises a patch of skin covered in nerve fibres that provide sensory to the corresponding spinal root (5).

This article provides a comprehensive overview of dermatomes, their anatomical basis, and their clinical significance in the sensory examination.

## History and Development of Dermatome Models

The history of dermatome mapping showcases the evolution of scientific exploration of the nervous system, starting with basic observations and leading to the creation of clinically essential tools.

Early studies, especially those conducted by Sir Henry Head in 1893, were crucial in identifying the segmental patterns of skin innervation based on the lesion distribution caused by herpes zoster (shingles) (6). Head’s dermatome maps, although limited by subjective data and variability among patients, were the first to illustrate the connection between spinal nerves and specific regions of skin sensation (7). These findings mark a crucial step in understanding the connection between nerve roots and sensory distribution, paving the way for significant advancements that followed.

Subsequent studies, especially those conducted by Otto Foerster in 1933, significantly advanced the field. Foerster employed a systematic approach that included rhizotomy, the surgical severance of nerve roots, to investigate sensory loss in individual dermatomes (8, 9). His work led to a more precise understanding of dermatomes, emphasising their overlap and variability (8). By systematically correlating sensory deficits with the affected spinal nerves, Foerster’s research provided strong evidence that became the foundation for modern dermatome maps (8). Later, in 1948, Keegan and Garrett refined these maps by examining the limb’s segmental distribution of cutaneous nerves (10).

Based on a literature systemic review by Lee et al. (11), Table 1 summarises the three standard dermatome maps and the quality of evidence based on the review. Two commonly used and accepted maps are the Keegan and Garrett map (10) and the Foerster map (3), the latter of which is featured in the American Spinal Injury Association scale of assessing spinal injury (12, 13).

Discrepancies in dermatome maps arise from methodological differences, population variability, and the complexity of sensory nerve distributions (4, 7). Early maps, like those by Head, relied on clinical observations, while later maps by Foerster, Keegan and Garrett used experimental approaches, leading to variations in defining boundaries. Genetic diversity and anatomical variations among populations investigated can yield different findings (7). Another discrepancy stems from the contrast between clinical and embryological perspectives. Embryological maps focus on the developmental origins of dermatomes from somites, while clinical maps are based on sensory loss among patients (11, 14). The overlap between dermatomes and sensory compensatory mechanisms further complicates the consistency of these maps.

According to Foerster (3), Keegan and Garrett (10), each dermatome overlaps with an adjacent dermatome. Dermatome overlap is an inherent feature of the nervous system and has evolutionary, anatomical, and functional origins. From an evolutionary perspective, overlap ensures redundancy in sensory innervation, reducing the risk of complete sensory loss if a single nerve root is damaged (11). Anatomically, the formation of nerve plexuses, such as the brachial and lumbar plexuses, contributes to the blending of fibres from adjacent spinal nerves. This anatomical intertwining results in the innervation of a single skin area by multiple nerve roots, particularly in regions like the trunk (15). Functionally, this overlap allows adjacent nerves to compensate for sensory input, maintaining sensation even when one nerve is compromised (16). This redundancy is vital for protecting the body from environmental hazards.

The extent of dermatome overlap is most evident in the trunk, where the sensory territories of adjacent nerves often blend extensively (4, 7). In contrast, overlap is less pronounced in distal areas like the hands and feet, where sensory territories are more distinct (4). This variation in overlap has significant clinical implications. For instance, the extensive overlap in the trunk can make it challenging to pinpoint the exact nerve root involved in a sensory deficit. In contrast, the less pronounced hand and foot overlap can provide more reliable diagnostic information.

Interindividual dermatome variation can occur due to intersegmental anastomoses of posterior spinal rootlets (17). This term refers to the situation when the sensory neurons of a dorsal root ganglion enter the spinal cord at a different level, leading to a potential overlap in the sensory territories of adjacent dermatomes (17, 18). Understanding the distribution of dermatomes is crucial for diagnosing and treating neurological and spinal cord injuries. The boundaries between respective dermatomes supplied by each nerve root could have been more precise due to some overlap between adjacent nerve territories (16). For example, sensory changes in the L5 dermatome may also reflect contributions from L4 and S1, complicating the identification of a specific lumbar nerve root lesion.

## Surface Anatomy and Key Examination Points

Dermatomes, the intricate components of the human nervous system, transmit sensory information from the skin. The distribution of dermatomes on the thorax and abdomen, in a striped pattern, and their dip inferiorly as they course from posterior to anterior present a fascinating complexity (17). However, the unique patterns of the upper and lower limb dermatomes, influenced by the early embryonic stages of limb development, captivate the imagination and add another layer of intrigue to their study (17).

To better comprehend the distribution of limbs dermatome, envision someone standing upright with their limbs abducted and thumbs pointing upwards. In this position, the dermatomes aligned as before the limbs rotated (18). It is essential to note that the innervation region of peripheral nerves is not equivalent to dermatomes (18). Peripheral nerves are derived from various plexuses, including brachial, lumbar, and sacral, which contain fibres from multiple spinal nerves (18).

**Figure 1:**
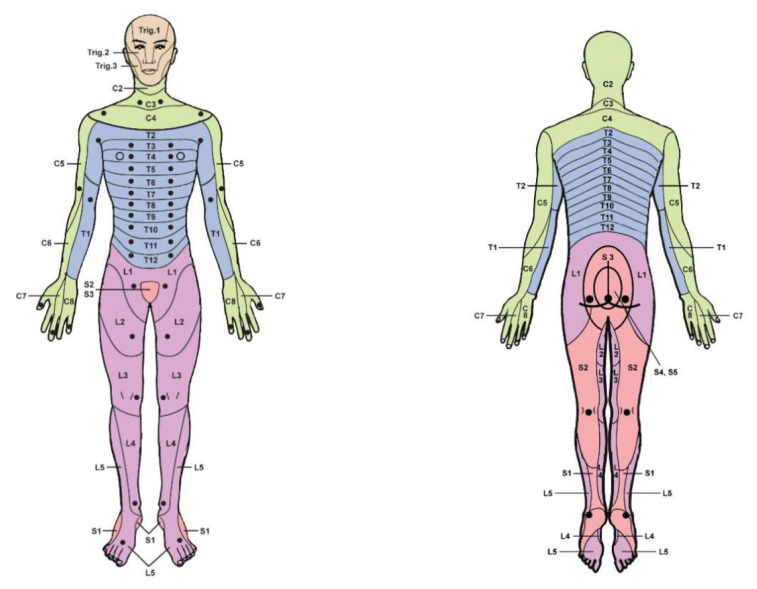
Schematic depiction of key points for sensory testing (anterior and posterior) Source: Adapted from American Spinal Injury Association (13)

Cervical 1 (C1) root does not have a dermatome to be tested (15). Dermatomal levels according to the Foerster map and the international standards booklet for neurological and functional classification of spinal cord injury have been summarised in Table 2 (13, 19) and illustrated in Figure 1.

Their reliability and safety underpin the committee’s recommendation of key sensory points. Each point corresponds to a specific dermatome identified in widely recognised anatomical references, providing a solid foundation for examination (13, 19). Furthermore, these points are associated with anatomically distinct bony landmarks, making them safe and easy to locate. This recommendation ensures consistency and reliability among examiners, instilling confidence and security in the process and the results (19).

**Figure 2:**
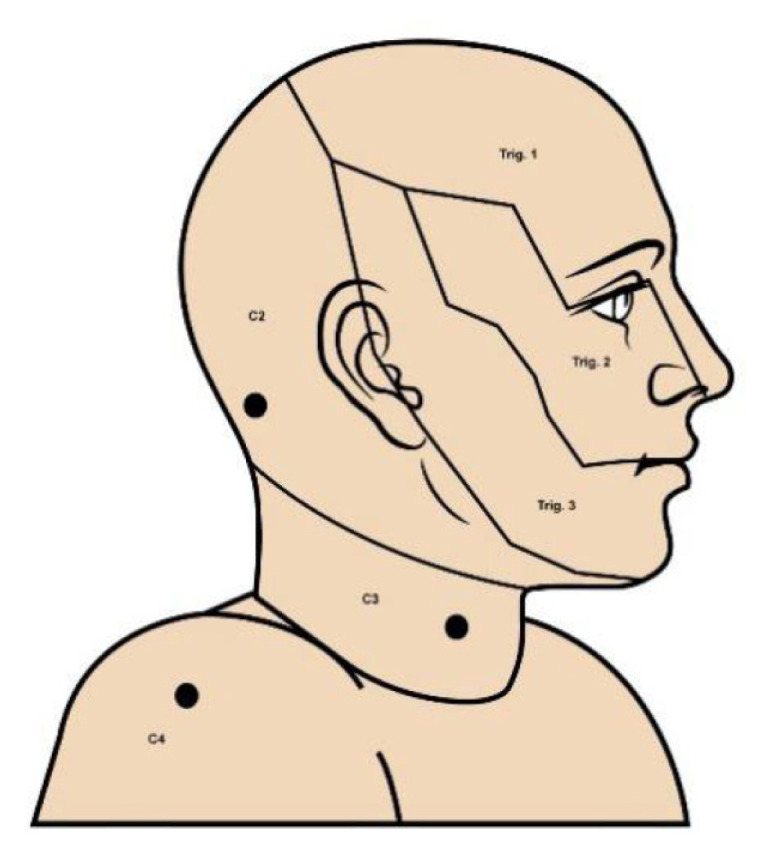
Schematic depiction of the lateral image of trigeminal sensory distribution and key examination points

Face sensory, on the other hand, is supplied by the trigeminal cranial nerve, which gives rise to three cutaneous branches: ophthalmic, maxillary, and mandibular nerve. Each branch of the trigeminal nerve supplies the face according to its distribution, as shown in Figure 2 (20). The key examination point of each dermatome can be described according to the plastic aesthetic unit, a concept in plastic surgery that divides the face into distinct regions based on the natural lines and contours of the face and based on the surface tension lines (21). Table 3 summarises the dermatome distribution of trigeminal nerve branches supplying the face and the key sensory examination points.

Despite these tools, the variability in sensory territories necessitates clinicians to adopt a comprehensive approach. Dermatome testing alone may not reliably localise lesions, underscoring the importance of complementary diagnostic tools such as imaging and electrophysiological studies. Magnetic resonance imaging can identify structural abnormalities affecting spinal nerves, while nerve conduction studies and somatosensory evoked potentials can confirm the functional integrity of sensory pathways. Together, these methods enhance the accuracy of diagnosis, highlighting the crucial role of medical professionals in ensuring conclusive clinical findings.

## Sensory Examination Technique

There are two types of sensation: exteroceptive and proprioceptive (22). Exteroceptive sensation, or superficial sensation, involves skin and mucous membrane receptors. It includes tactile or touch sensation, pain sensation, and temperature sensation. The segmental distribution of proprioceptors does not follow the dermatomal map but is closely associated with the muscle innervation pattern (22).

The spinal nerve root sensory level can be graded using the American Spinal Injury Association chart (19), a standardised tool for assessing sensory and motor function in patients with spinal cord injury. The chart assigns a grade to the sensory level according to the relevant dermatome described in Table 2 or Figure 1.

Grading scale for sensation (with comparison to the sensation on the patient’s reference point):

0: Absent, no response
1: Altered (impaired or partial appreciation, including hyperesthesia)
2: Normal or intact (similar to reference point)
NT: Not testable

Sensory examination is a crucial neurological assessment that requires the patient’s optimal cooperation in a comfortable environment (2). The examiner should explain each procedure and what is expected from the patient. Before starting, the patient must be asked if they have experienced abnormal sensations, numbness, or pain in any part of their body. Establish a baseline by ensuring the patient has normal sensation on the forehead or sternum (reference point) and whether they can feel the test object as soft or sharp (22).

Pain examination begins with demonstrating the test on the forehead as a reference point with a neurotip or neurological pin. The examiner then gently pricks the skin, maintaining even pressure over each dermatome C2–S5 bilaterally on the key sensory examination point while the eyes are closed or vision is blocked. The patient’s feedback is crucial, as they should indicate whether the sensation is equal, increased, decreased, or absent compared to the forehead.

The examination was then repeated to assess soft touch using a wisp of cotton wool or 10g Semmes-Weinstein monofilament and temperature using two test tubes with stoppers: one be filled with cold water (between 5°C and 10°C) and the other with warm water (40°C to 45°C). This temperature range ensures an accurate assessment of temperature sensation without overlapping with pain perception, making it a reliable tool for temperature testing in neurological examinations. Examination of two-point discrimination would be conducted by repeating similar steps using a two-point discrimination aesthesiometer.

A detailed step-by-step guide for sensory examination, including visual aids and the rationale for using the respective assessment tools, has been covered by Khoo et al. (22). Please refer to the YouTube videos: https://youtu.be/dcPzgx5kzjY and https://youtu.be/5EhlG9l2wHo

## Conclusion

Dermatome and sensory examination are not just tasks but are crucial in assessing and diagnosing various medical conditions. An appropriate examination technique is essential to lessen the disparity between examiners. We hope medical trainees and staff can use this method for neurological assessments.

The link to the dermatome anatomy examination video, a valuable resource for your learning, is readily available at the following link: https://youtu.be/7frtOoeppuo

## Figures and Tables

**Table 1:** Review of dermatomal map

Source	Methodology used	Quality of evidence
Keegan and Garrett’s map (10)	Distribution of cutaneous sensory impairment after intervertebral disc prolapse in humans	Intermediate
Foerster map (3)	Isolation of a single dorsal nerve root by surgical section of several (at least 2) adjacent dorsal nerve roots above and below and mapping of the residual area of cutaneous sensation in humans	Good
Head and Campbell map (11)	Distribution of skin erythema and blistering after herpes zoster reactivation (shingles) in humans, together with histologic confirmation of dorsal root ganglion level	Good

**Table 2:** Dermatomal level with key sensory points

Dermatome	Distribution	Key sensory examination point
C2	Posterior aspect of the head (including the angle of mandible)	At least 1 cm lateral to the occipital protuberance (alternatively 3 cm behind the ear)
C3	Anterior neck, posterior aspect of upper neck and head, and supraclavicular fossa	Supraclavicular fossa (posterior to the clavicle) and at the midclavicular line
C4	Shoulder and skin of infraclavicular fossa and posterior lower neck	Over the acromioclavicular joint
C5	Lateral aspect of the upper extremities at and above the elbow	Lateral (radial) side of the antecubital fossa just proximal to the elbow crease
C6	The forearm and radial side of the hand	Dorsal surface of the proximal phalanx of the thumb
C7	Central aspect of posterior forearm and middle finger	Dorsal surface of the proximal phalanx of the middle finger
C8	Ulnar side of the forearm and hand and little finger	Dorsal surface of the proximal phalanx of the little finger
T1	Extends to the medial aspect of the forearm and distal arm	Medial (ulnar) side of the antecubital fossa, just proximal to the middle epicondyle of the humerus
T2	Medial and proximal aspects of the arm continuing into the axilla	Apex of axilla
T3	Anteriorly and posteriorly at the level of the lower axilla	Midclavicular line and third intercostal space
T4	Anteriorly and posteriorly at the level of the nipple; In between the fourth and fifth rib	Midclavicular line and at the level of the nipple line
T5	Anteriorly and posteriorly at the level just inferior to the nipple; In between the fifth and sixth rib	Midclavicular line and midway between T4 and T6
T6	Anteriorly and posteriorly at the level of the xiphoid process; In between the sixth and seventh rib	Midclavicular line and at the level of xiphisternum
T7	Evenly distributed anteriorly and posteriorly between T6 and T8 dermatomes	Midclavicular line and midway between T6 and T8
T8	Evenly distributed anteriorly and posteriorly between T7 and T9 dermatomes	Midclavicular line and midway between T6 and T10
T9	Evenly distributed anteriorly and posteriorly between T8 and T10 dermatomes	Midclavicular line and midway between T8 and T10
T10	Anteriorly and posteriorly at the level of the umbilicus	Midclavicular line and at the level of the umbilicus
T11	Evenly distributed anteriorly and posteriorly between T10 and T12 dermatomes	Midclavicular line and midway between T10 and T12
T12	Anteriorly just superior to the pelvic girdle	Midclavicular line and midpoint of Inguinal ligament
L1	Posteriorly includes the skin lateral to the L1 vertebra and wraps anteriorly to the groin and pelvic girdle area overlying the inguinal canal	Midway distance between the key sensory points for T12 and L2
L2	Anteriorly covers the thigh inferior to the inguinal canal	On the anterior-medial thigh at the midpoint drawn on an imaginary line connecting the midpoint of the inguinal ligament and the medial femoral condyle
L3	Evenly spaced between L2 and L4, extending down the medial aspect of the thigh and leg	Medial femoral condyle above the knee
L4	Anteriorly curves from the lateral aspect of the thigh to the medial aspect of the leg and foot; Includes the knee, medial surface of the big toe, and medial malleolus	Medial malleolus
L5	Posterolateral aspect of the thigh wrapping anteriorly at the level of the knee to cover the anterolateral aspect of the leg; Includes the dorsal and plantar aspects of the foot, lateral surface of the big toe, and toes 2, 3, and 4	Dorsum of the foot at the third metatarsal phalangeal joint
S1	From the sole and plantar surface of the toes, spreads upwards along the posterior side of leg	Lateral aspect of the calcaneus
S2	Posterior surface of thigh and leg	Midpoint of the popliteal fossa
S3	The skin over the genital region of male and female, respectively	Ischial tuberosity or infragluteal fold
S4	Perineal region	Perianal area less than 1 cm lateral to the mucocutaneous junction (taken as one level)
S5	Skin adjacent to the anus	

Notes: C = cervical; L = lumbar; S = sacral; T = thoracic

**Table 3:** Face dermatome level with key sensory points

Trigeminal nerve branch	Distribution	Key examination point
Ophthalmic nerve	Tip of nose to the scalp (vertex) along upper eyelid and forehead	Point of intersection between superior forehead wrinkle line and mid-pupillary line FCU: Central subunit of the forehead
Maxillary nerve	The area from the lower eyelid to the upper lip and the buccae	Mid-pupillary line below the lower eyelid FCU: Infraorbital subunit of cheek
Mandibular nerve	Area from the lower lip to the lower part of the mandible and parts of the external ear	Mid-pupillary line over the body of the mandible FCU: Inferior portion of the buccal subunit of cheek

Note: FCU = facial cosmetic unit
